# A Novel Immunofluorescent Computed Tomography (ICT) Method to Localise and Quantify Multiple Antigens in Large Tissue Volumes at High Resolution

**DOI:** 10.1371/journal.pone.0053245

**Published:** 2012-12-31

**Authors:** Geraint J. Parfitt, Yilu Xie, Korey M. Reid, Xavier Dervillez, Donald J. Brown, James V. Jester

**Affiliations:** The Gavin Herbert Eye Institute, University of California Irvine, Irvine, California, United States of America; Charité Universitätsmedizin Berlin, NeuroCure Clinical Research Center, Germany

## Abstract

Current immunofluorescence protocols are limited as they do not provide reliable antibody staining within large tissue volumes (mm^3^) and cannot localise and quantify multiple antigens or cell populations in the same tissue at high resolution. To address this limitation, we have developed an approach to three-dimensionally visualise large tissue volumes (mm^3^) at high resolution (<1 µm) and with multiple antigen labelling, for volumetric and quantitative analysis. This is made possible through computer reconstruction of serial sectioned and sequentially immunostained butyl-methyl methacrylate (BMMA) embedded tissue. Using this novel immunofluorescent computed tomography (ICT) approach, we have three-dimensionally reconstructed part of the murine lower eyelid that contains the meibomian gland and localised cell nuclei (DAPI), Ki67 and cytokeratin 1 (CK1), as well as performing non-linear optical (NLO) microscopy imaging of collagen, to assess cell density, cell proliferation, gland keratinisation and gland volume respectively. Antigenicity was maintained after four iterative stains on the same tissue, suggesting that there is no defined limit to the number of antigens that can be immunostained for reconstruction, as long as the sections remain intact and the previous antibody has been successfully eluted. BMMA resin embedding also preserved fluorescence of transgenic proteins. We propose that ICT may provide valuable high resolution, three-dimensional biological maps of multiple biomolecules within a single tissue or organ to better characterise and quantify tissue structure and function.

## Introduction

Recent advances in optical microscopy and digital image processing has greatly expanded our ability to visualise cellular structure and function. Non-invasive, optical sectioning of thick, intact tissues using confocal microscopy has paved the way for more accurate three-dimensional reconstruction of biological systems [1]. Advances in non-linear optical imaging and two-photon microscopy has led to improvements in optical depth penetration using near-infrared light with reduced cell toxicity and photo-bleaching to improve live cell imaging *in vitro* and *in vivo* [2], [3], [4]. More recently, stimulated depletion emission (STED) has improved lateral and axial resolution to surpass the diffraction limit and provides single molecule detection [5], [6], [7]. The re-introduction of single plane illumination microscopy (SPIM) with wide-field imaging combined with high-speed camera detection has extended our temporal resolution in detecting rapid cellular and intracellular events, as well as our ability to image large scale structures and probe events during development [8], [9].

By contrast, immunohistochemistry, first introduced by Albert Coons et al in the 1940s [10], [11], has remained, for the most part, restricted to the simultaneous localisation of a limited number of antigens in two-dimensional tissue sections or small tissue volumes. As a result, quantitative analysis of different cell types and their respective densities is problematic and suffers from sampling error; plus, no large-scale three-dimensional volumetric data can be coupled with current immunostaining techniques. Whole mount staining and confocal microscopy provide three-dimensional information, however, antibody penetration is unreliable through large tissues (>0.3 mm^3^) and the imaging depth is limited by attenuation of light and the working distance of the objective. Light-sheet microscopy removes the problem of an objective’s working distance and provides high-resolution deep tissue imaging, on the centimetre scale [9]. However, light sheets are optimally used for detection of transgenically expressed fluorophores, such as GFP, as whole mount immunostaining is subject to the same limitations found using confocal microscopy. Whole mount preparations also typically involve structural manipulation of the tissue for appropriate viewing under the microscope, but are not subject to potential shrinkage artefacts that may occur with resin embedding for histological analysis when dehydrated with ethanol and/or thermal polymerisation. Two-photon imaging can visualise tissue depths of over 1 mm [3], [4], but also requires whole mount staining if immunofluorescent imaging is desired. Finally, the axial resolution of confocal microscopy is rarely better than 700 nm, except when using STED [6], [7], and is lower in comparison to imaging thin physical sections individually.

While cryo-sectioning or embedding of specimens in paraffin wax or agarose provides uniform immunostaining in a two-dimensional plane, blocks are difficult to cut serially and sections are challenging to treat repeatedly with solutions while maintaining tissue structure. These techniques are therefore more suited to high-throughput testing and diagnostic analysis as opposed to highly detailed mapping of antigens on a whole tissue scale, which is considerably more labour intensive. Hard polymer resins may be used (i.e. LR White, methyl methacrylates and araldite) as an alternative for superior structural preservation of tissue that facilitates thinner sectioning for higher z-axis resolution and serial sectioning. Recently, an array tomography technique [12] described three-dimensional reconstruction of small tissue volumes with high resolution and multiple, iterative antibody labelling using LR White resin as the embedding medium. Using this staining protocol, antibody penetration is limited in thicker sections (over 200 nm) and antigens may only be stained superficially [13] as LR White may not be readily removed with an organic solvent. Thermal curing at high temperatures is often required for LR White polymerisation which can also result in further tissue shrinkage and significantly lowered antigenicity. Thus, LR White is generally not considered a good candidate for immunohistochemistry studies [14].

To develop a novel immunofluorescent computed tomography (ICT) method that overcomes the immunolabelling limitations described above, we evaluated using butyl-methylmethacrylate (BMMA) as the embedding medium. BMMA has routinely been used for histological analysis over many years [15], [16] and is a versatile polymer that can be formulated with a variable hardness to suit tissue composition and may be cured at low temperatures under UV light [16], [17]. Although BMMA is not commonly used for serial sectioning and reconstruction of tissues, it may easily be sectioned at thin (100–500 nm) and semi-thin (0.5–5 µm) thicknesses. In this paper, we show that antibody probes fully penetrate semi-thin BMMA sections and can then be stripped from serial sections, allowing different antigen targets to then be three-dimensionally reconstructed on the whole tissue scale. Conveniently, this approach may be performed with a conventional fluorescent microscope fitted with an automated stage for montage imaging. Physical sectioning of tissue also allows for greater z-axis resolution than confocal microscopy and more consistent staining over large volumes than *en bloc* staining techniques. This technique can be coupled with other imaging modalities and may theoretically be applied to quantify any number of cell populations and markers in any fixed tissue.

## Results

### Antibody Staining of Single BMMA Tissue Sections

**Figure 1:**
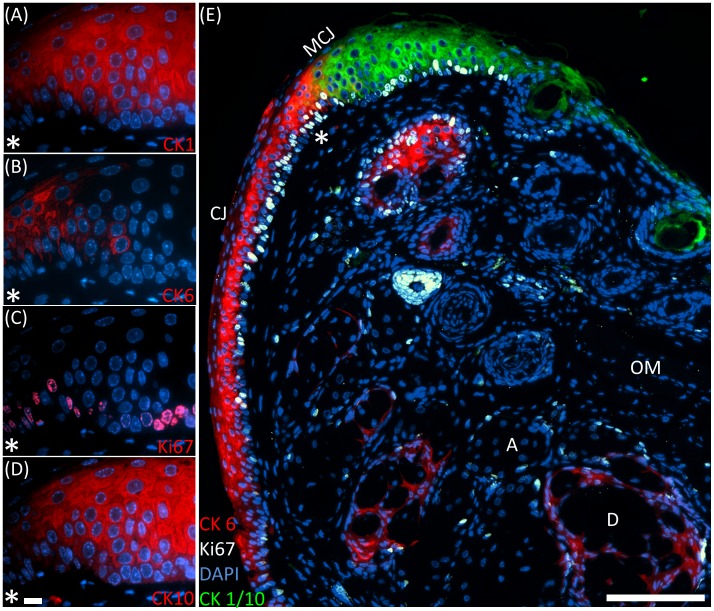
Iterative antibody labelling of a BMMA embedded 2month old mouse eyelid. (A) Anti-CK1– Eyelid margin. (B) Anti-CK6– palpebral conjunctiva. (C) Anti-Ki67– nuclei of proliferating cells. (D) Anti-CK10– acidic cytokeratin which co-localises with the basic CK1 at the epidermis. (E) Reduced resolution overlay of (A, B, C, D) illustrating multiple stains (four immunolabels and one counter-stain) on the same section at a thickness not previously possible. Scale bars = 10 µm (A), 100 µm (B). OM = Obicularis Muscle, A = Acinus, CJ = Conjunctiva, MCJ = Mucocutaneous Junction, D = Duct.

To demonstrate iterative antibody staining, a single 2 µm section of a 2month old mouse meibomian gland was sequentially probed for the filamentous proteins cytokeratin (CK) 1, 6 and 10 as well as the cell proliferation marker Ki67. Immunolabelling of CK1 (Fig. 1A) visualised keratinisation of the eyelid margin and showed staining of the epidermis (Fig. 1E). Following elution of both primary and secondary antibodies with glycine hydrochloride pH 2.0, subsequent immunolabelling with anti-CK6 (Fig. 1B) showed staining of the conjunctiva (CJ) up to the eyelid margin (Fig. 1E). Successful elution of the prior antibody was demonstrated by the lack of cross-labelling between CK1 and CK6 staining with the same anti-rabbit Alexa Fluor 546 secondary antibody probes. The abrupt transition from CK1 staining to CK6 staining at the eyelid margin is also consistent with the transition of keratinised epidermis to non-keratinised conjunctiva at the mucocutaneous junction (MCJ). CK6 labelling (Fig. 1B) was also present in the meibomian gland ductal epithelium (Fig. 1E, D). After stripping of anti-CK6 probes, the localisation of Ki67^+^ nuclei was evaluated through anti-Ki67 staining (Fig. 1C). Cells which are not in G_0_ phase and are actively cycling express Ki67 protein in the nucleus and therefore, Ki67 is considered a marker for cellular proliferation. In the 2month old mouse, cells within a hair follicle as well as the basal cells of the meibomian gland acini and epithelium were seen to stain intensely for Ki67, indicative of cell proliferation (Fig. 1E). Again, no cross-labelling was observed in the palpebral conjunctiva where Ki67^+^ nuclei are also present, demonstrating a complete stripping of previous CK 6 primary antibodies. Finally, the sections were probed for CK10 (Fig. 1D), which co-localises with CK1 (compare Fig. 1A with D). As cytokeratins are present as acid-base pairs, we used the acidic CK10 pair of CK1 to illustrate that sequential stains may be used to demonstrate co-localisation with no loss of signal intensity.

**Figure 2:**
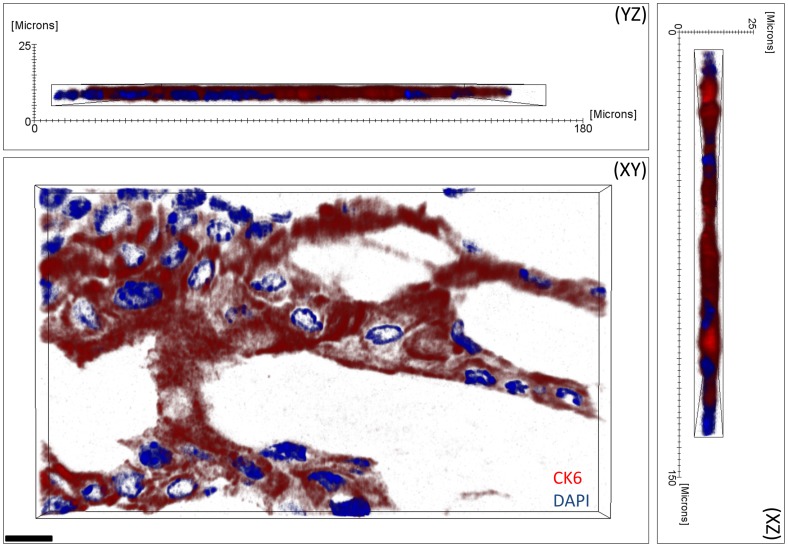
Confocal microscopy three-dimensional reconstruction of a 2 µm thick BMMA tissue section from the mouse meibomian gland stained with anti-CK6 (red) and DAPI (blue). Antibody penetration is observed throughout the entire 2 µm section for anti-CK6 and DAPI. Scale bar = 10 µm.

For each labelling step, only anti-rabbit Alexa Fluor 546 was used to demonstrate that the primary antibody of the previous labelling had been fully removed, as detailed above. No cross-labelling was observed at each staining step (Fig. 1E). Additionally, confocal microscopy of a single 2 µm section immunostained for CK 6 (Fig. 2) revealed that antibodies are able to penetrate through the whole section depth evenly and therefore, large volumes may be reliably stained and reconstructed for the quantification and localisation of antigens using BMMA embedding. The concentration of both primary and secondary antibodies may also be significantly reduced when compared to cryo or paraffin section immunostaining as the sections are cut at 2 µm. For image acquisition, exposure times were set at 500 ms for Alexa Fluor 546 and 25 ms for DAPI. No photo-bleaching was observed as sections were only imaged once, and because of its known robustness under intensive imaging [18], anti-rabbit Alexa Fluor 546 was the only conjugated fluorophore used in this experiment.

### Three-Dimensional Volume Reconstruction

**Figure 3:**
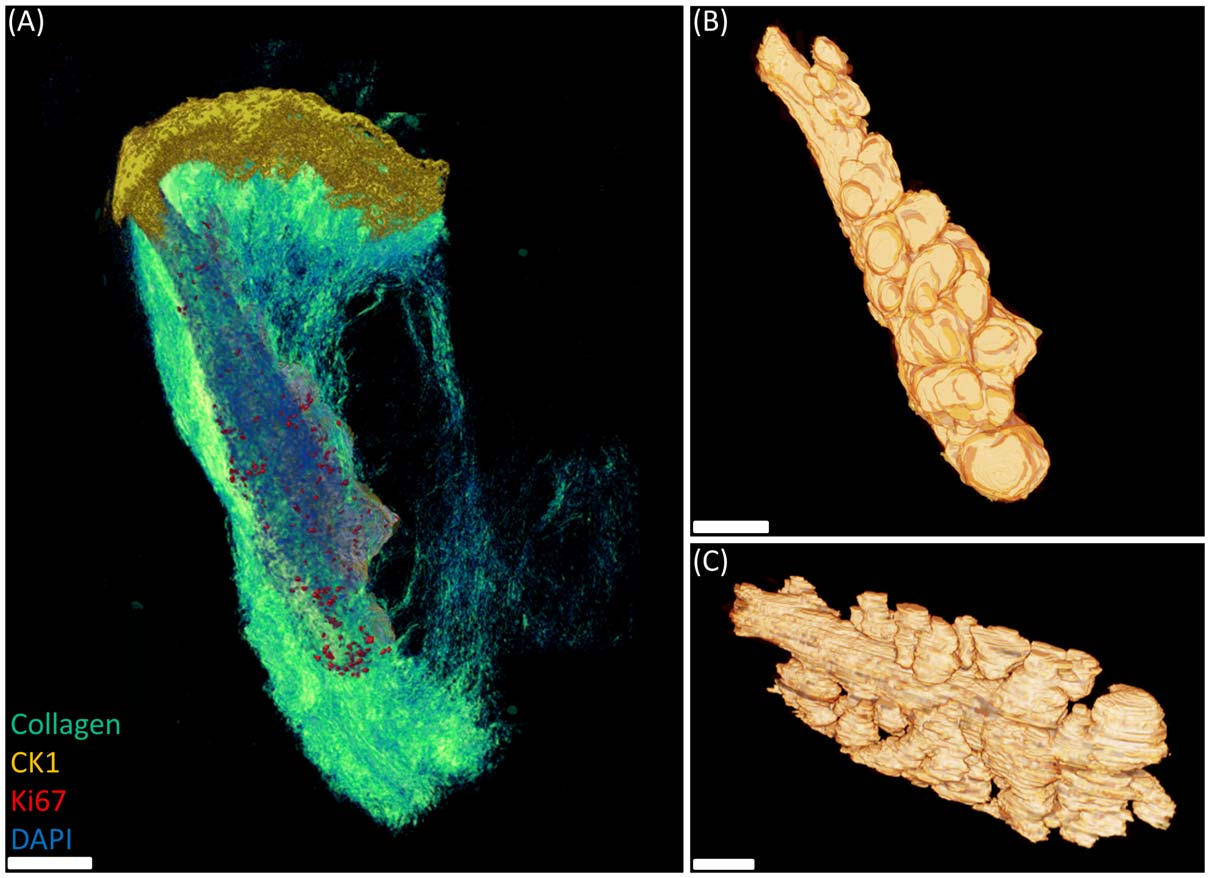
Three-dimensional reconstruction of a 2 year old mouse eyelid by ICT and NLO-SHG imaging. (A) Cytokeratin (yellow) aligns the eyelid margin until the mucocutaenous junction while Ki67 (red) distribution is limited to basal cell nuclei in the ductal epithelium and acini. (B and C) Segmented reconstruction of the mouse meibomian gland. Scale bars = 50 µm.

The full ICT approach, utilising both non-linear optical (NLO) microscopy and fluorescence imaging modalities with iterative antibody multilabelling, was used to reconstruct a 2 year old mouse eyelid. From this, it was possible to identify and localise cell proliferation in the meibomian gland simultaneously with keratinisation at the ductal orifice and eyelid margin. After second harmonic generation (SHG) imaging to map the collagenous extra-cellular matrix of the tarsal plate (Fig. 3A, cyan), sections were immunostained for Ki67 to identify cells undergoing cell cycling and imaged accordingly. Antibodies were then eluted from the sections with 0.1% sodium dodecyl sulphate (SDS) and subsequently stained with anti-CK1, and each individual section was again imaged at the same resolution. To reconstruct the tissue, individual images representing each section as a two-dimensional plane were aligned and registered semi-automatically, and then rendered using Amira software. The whole meibomian gland (Fig. 3B and C) was segmented and reconstructed three-dimensionally from the tarsal plate using the collagen-SHG image mask (see Movie S1). From the known voxel size, it was possible to calculate the volume of the meibomian gland and individual acini (Table 1). The 2 year old mouse meibomian gland had a total volume of 10.03×10^6^ fl (1 femtolitre = 1 µm^3^), while single acini ranged from 0.004–0.282×10^6^ fl.

**Table 1:** Quantitative analysis of the 2 year old mouse meibomian gland by ICT.

	Volume (flx10^6^)	Proportion of cells cycling		
	1 fl (femtolitre) = 1 µm^3^	Total Nuclei	Ki67^+^ Nuclei	
			(n)	%
**Meibomian Gland**	10.03	9126	834	9.1%
**Acinus**				
**1**	0.004	97	13	13.4%
**2**	0.054	184	32	17.4%
**3**	0.094	148	29	19.6%
**4**	0.110	156	25	16.0%
**5**	0.089	149	51	34.2%
**6**	0.098	193	44	22.8%
**7**	0.189	354	63	17.8%
**8**	0.235	165	31	18.8%
**9**	0.282	492	114	23.2%
**10**	0.265	477	89	18.7%
**Mean**	0.142	242	49	20.2%
**SD (±)**	0.095	144	32	5.7%

**Figure 4:**
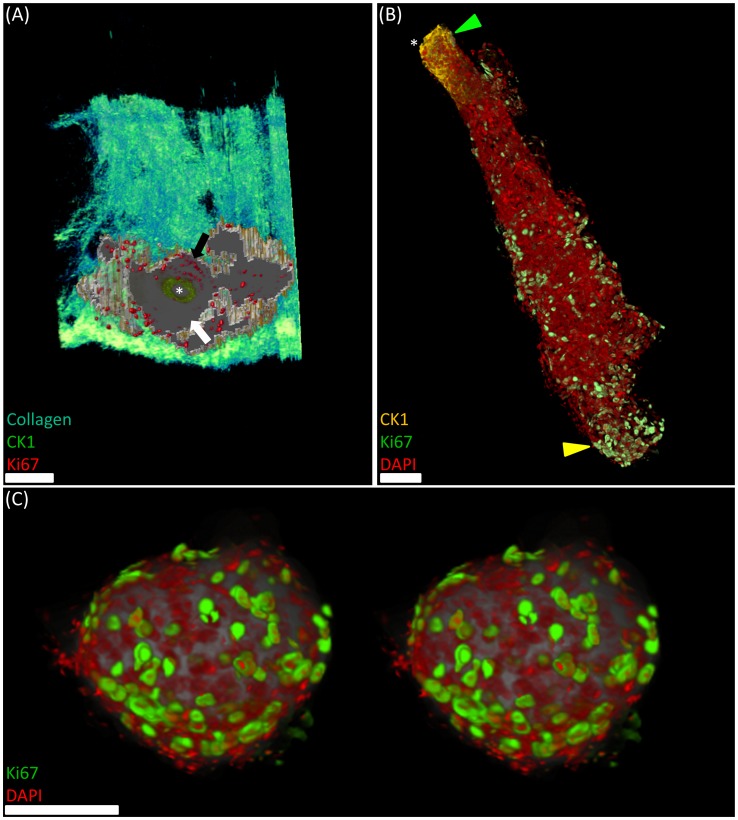
Immunofluorescent computed tomography (ICT) imaging of a 2 year old mouse eyelid and meibomian gland. (A) Transverse view of the meibomian gland three-dimensional reconstruction from the distal region of the duct. Ki67 positive nuclei are localised to the ductal epithelium farthest from the conjunctiva (black arrow) with cytokeratin 1 labelling illustrating the ductal orifice (asterisk). (B) Three-dimensional reconstruction of CK1 (green arrowhead), Ki67 (green) and DAPI labelling (red) in the whole meibomian gland and individual acini (yellow arrowhead). (C) Stereo-pair of a single acinus with ki67 positive basal cell nuclei. Scale bars = 50 µm.

From the three-dimensional reconstructions, we can qualitatively confirm the presence of CK1 penetrating the ductal orifice of the meibomian gland (Fig. 4A asterisk and B yellow arrow, also Movie S2). Also, Ki67 positive cells appeared to form clusters at distinct regions within the eyelid and meibomian gland. We observed a high cell cycling count lining the ductal epithelium adjacent to the orbicularis muscle (Fig. 4A, black arrow) and also in the basal cells of each acini, particularly in the most distal acinus (Fig. 4B, green arrow). The basal cells of the ductal epithelium that lie adjacent to the conjunctiva appear to show almost no cell cycling (Fig. 4A white arrow). To observe the localisation of Ki67^+^ basal cells in the meibomian gland, the reader is referred to Movie S3. In the whole meibomian gland volume there were 8225 cell nuclei counted, of which, 801 expressed Ki67 and were therefore in some stage of active cell cycling. Single acini were also quantified and displayed a mean cell cycling percentage of 19.2±3.2% (Table 1).

**Figure 5:**
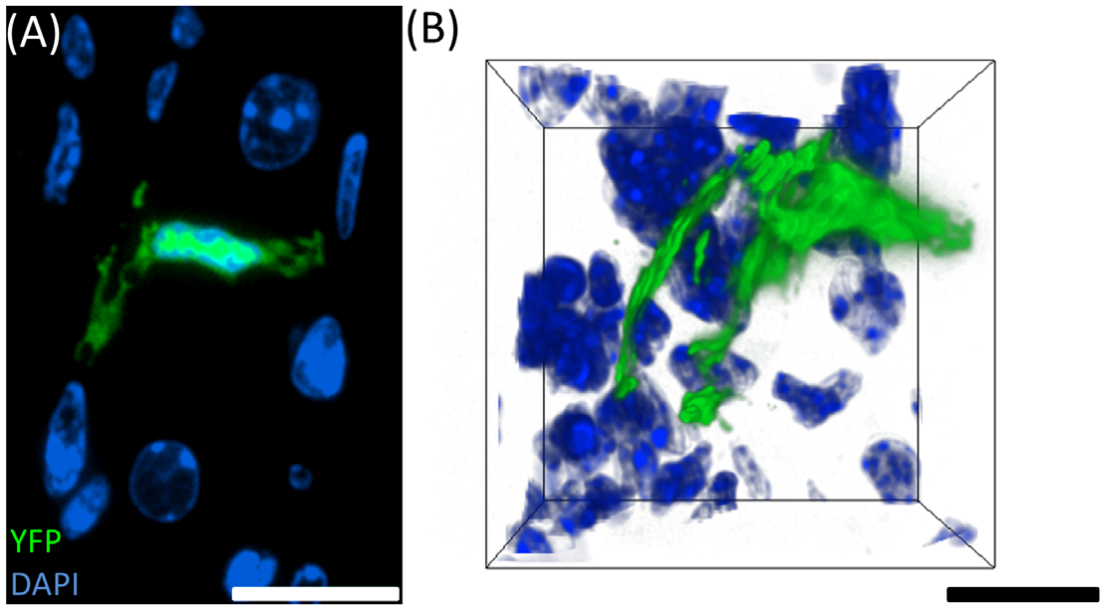
Three-dimensional reconstruction of an eYFP^+^ cell after BMMA processing for ICT. (A) Cross-section of a dendritic cell in the eYFP-CD11c Mouse trigeminal ganglion. (B) Three-dimensional reconstruction (20 µm volume) of an eYFP^+^ DC in the mouse TG. Scale bars = 10 µm.

To determine whether eYFP, an analogue of GFP, is maintained post-processing in ICT, an eYFp-CD11c mouse trigeminal ganglion was visualised with fluorescence microscopy after BMMA sectioning at 500 nm (Fig. 5A). Indeed, eYFP was retained in the dendritic cells after BMMA processing and can be easily observed in both thin (500 nm) and thick (2 µm) sections at low magnifications, further expanding the capabilities of ICT. The three-dimensional volume in Fig. 5B depicts an eYFP^+^ dendritic cell in the mouse trigeminal ganglion (TG) with dendritic extensions traversing the gap in-between cell nuclei.

## Discussion

Through ICT, we have demonstrated a method for highly specific localisation and quantification of multiple antigens and cell populations in a large tissue volume that, in theory, can cover entire organs. ICT incorporates high-resolution macroscopy (HR-Mac) [19], [20], [21] with sequential antibody staining to enable structural, volumetric and densitometric analysis, as well as quantification of multiple, specific target molecules three-dimensionally with high specificity and at microscopic resolution. From the ICT three-dimensional reconstruction in Fig. 3, we can observe CK1 expression three-dimensionally while quantifying the extent and location of meibocyte proliferation in a 2 year old mouse meibomian gland. Comparative analysis with younger mice will allow us to more accurately determine the age-related changes that occur in the meibomian gland and whether they contribute to dysfunction of the gland and consequently, dry eye related disorders.

There are significant advantages of ICT when compared to routine immunofluorescent staining procedures; physical BMMA sectioning means tissue morphology is well preserved, axial resolution is high and antibody staining is consistent over a large volume. Antigenicity is well preserved because of the low temperature polymerisation, which is also likely to minimise tissue shrinkage artefacts known to result from thermal curing. It is also possible to repetitively perform antigen retrieval on BMMA serially sectioned tissue to potentially remove cross-links formed when fixing tissue with paraformaldehyde; further improving antigenicity. As BMMA may be removed with acetone, it is also easy to stain and readily remove antibodies for further re-probing, while antibody penetration is consistent through thicker sections. This iterative, multilabelling approach offers huge benefits, particularly for co-localisation studies where many antigens could be localised and quantified in the same tissue and may help identify cell-cell interactions between distinct populations. Moreover, as eYFP and its analogues may be used with ICT, it is possible to determine antigen interactions with identifiable cells. In theory, consecutive antibody staining and multiplexing could be limitless provided that the previous primary antibody has been dissociated from the epitopes with minimal tissue distortion. However, sections are more vulnerable to drying out and being damaged through repeated processing. It is also envisaged that ICT may be incorporated with other techniques for improved imaging and quantification, such as correlative scanning electron microscopy [12], [22] and in-situ hybridisation [14], [23], as well as SHG. Theoretically, ICT may be coupled with STED microscopy for increased lateral resolution, but the sizes of the data sets would be greater and more difficult to process for whole tissues and so it may be more feasible to localise a smaller region for reconstruction using this technique. ICT is also relatively inexpensive as only a standard fluorescent microscope with an automated stage is required for image acquisition.

In the current study, ICT visualised CK1 distribution at the mouse eyelid margin demarcating the mucocutaneous junction and extending into the meibomian gland ductal orifice, as previously shown two-dimensionally [24], [25],. It has been hypothesised that keratinisation of the ductal orifice plays a role in age-related meibomian gland dysfunction through blockage of lipid secretion onto the ocular surface [26], [27]. Quantitative assessment of a younger mouse eyelid CK1-ICT reconstruction will help determine whether there are significant differences in ductal keratinisation between young and old meibomian glands. The reconstruction also showed that Ki67^+^ cells are present in both the basal acini and ductal epithelial cells and account for 9.7% of the total cell population in the whole meibomian gland. In contrast, acini from the same meibomian gland exhibit approximately 19.2±3.2% of their total cell population in an active cell cycle phase. This proliferation rate most likely reflects the continual demand for lipids on the ocular surface to inhibit tear evaporation, which are provided predominantly through holocrine lipid secretion by the meibomian gland. It has previously been suggested that a decrease in cell proliferation rates with age in the meibomian gland may lead to decreased synthesis and deposition of lipids onto the eye [28], [29]; ICT could prove valuable in accurately determining whether this is the case.

In conclusion, ICT is a powerful new method for mapping antigens on both a micro and macroscopic scale that may aid our understanding as to aging, disease states, stem cell niches, DiO-labelled stem cell differentiation and motility after transplantation, developmental signalling and also co-localisation studies of immune cells upon viral infection, furthering the volume capacity of immunostaining beyond array tomography. While larger data sets are incurred, modern computing allows manipulation of these large datasets with relatively inexpensive hardware. ICT offers the ability of quantifying cell type and density in a whole tissue volume and can provide morphometric analysis through SHG and fluorescence imaging. ICT furthers the immunostaining capacity in a large volume through serial sectioning of semi-thin sections with no advancement or alteration in the microscopy and thus, improves axial resolution and structural integrity when compared to thicker cryo-sections. Also, ICT can be tailored to the requirements of the tissue and imaging modality; the block’s hardness may be modified to accommodate different tissue compositions and different thicknesses may be cut for immunostaining. Most importantly, BMMA offers morphologic preservation superior to that of paraffin or cryo-sections while maintaining antigenicity through low temperature polymerisation, and because of its easy removal, thicker sections may be reliably stained to provide an excellent platform for three-dimensional reconstructions, which localise and quantify specific cell populations in the same tissue.

## Materials and Methods

### Sample Processing

Animals were treated in accordance with the ARVO statement for the use of animals in ophthalmic and vision research and all procedures were approved by the IACUC of the University of California, Irvine (P.I. Jester, protocol# 2011-3002, approved September 8, 2011). Lower eyelids from a 2month and 2 year old C57BL/6 mouse were excised, and the tarsal plate and meibomian glands were further trimmed and fixed overnight in 2% paraformaldehyde in phosphate buffered saline (PBS). Blocks were then embedded in low melting point agarose (3%) the next day to preferentially orientate the tissue. Also, eYFP-CD11c mice [30] were euthanised and the TG excised from the brain to ascertain whether eYFP fluorescence is maintained in ICT. The TG was fixed overnight in 4% paraformaldehyde in PBS.

To formulate BMMA, butyl methacrylate and methyl methacrylate monomer solutions (Polysciences, Warrington PA) were mixed 1∶4, which yields a soft block necessary for softer tissues. Increasing the proportion of methyl methacrylate polymerises a harder block, which is more suitable for denser tissues as well as ultra-thin sectioning and electron microscopy. The reducing agent dithiothreitol, deemed to preserve antigenicity [16], was then added to yield a final concentration of 5 mM. The photo-initiator, benzoin ethyl ether (Sigma Aldrich, St Louis MO) was also added (0.3% of total volume), and the final BMMA solution was degassed with N_2_ for 30 minutes to remove oxygen which hinders polymerisation. The final BMMA solution was kept at −20°C to prevent heat and ambient light from prematurely polymerising the solution.

All specimens were dehydrated in 50%, 70% then 95% ethanol for 30 minutes each at room temperature before a final rotation in 100% ethanol for a further 30 minutes. After dehydration, samples were infiltrated with BMMA resin at 4°C, over three days, displacing the ethanol in an ascending BMMA concentration gradient from 25% to 75% BMMA in ethanol. The tissue was then submerged and rotated in 100% BMMA for 24 hrs at 4°C before the polymerisation stage. Each sample was subsequently placed in a BEEM gelatin capsule with fresh BMMA and sealed tightly with the smallest air bubble possible as this prevents polymerisation. For polymerisation, the capsules were placed under UV light for 20 hrs in a Pelco UCV2 UV cryo chamber (Ted Pella, Redding CA) set at 4°C.

### Serial Sectioning

**Figure 6:**
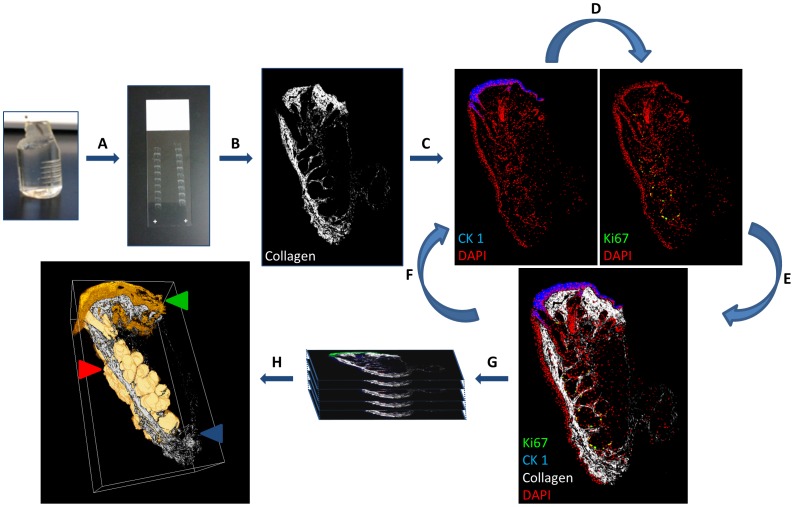
Stepwise Immunofluorescence Computed Tomography (ICT) protocol coupled with non-linear optical microscopy. (A) Serial section BMMA embedded tissue block (50 nm–5 µm). (B) SHG imaging visualises fibrillar collagen. (C) Immunofluorescent labelling and subsequent imaging. (D) Strip antibodies with glycine hydrochloride (pH 2.5) accordingly and re-stain to visualise multiple antigens in the same tissue sections. (E and F) Overlay images or repeat iterative antibody labelling. (G) Align and reconstruct. (H) Quantitative and qualitative analysis of cell populations may then be performed from 3-D reconstructions. (Red arrow: Meibomian gland segmented volume, Green arrow: CK1, Blue arrow: collagen SHG signal orthoslice).

Sections were serially cut using a diamond knife (DiAtome, Hatfield, PA) on a Reichert Ultracut R ultramicrotome. An adhesive (Pattex - Henkel, Dusseldorf, Germany) was added to the top and bottom of the block to bind each section, forming a ribbon that was floated onto a gelatin-coated slide. Chloroform vapour was also used to expand the sections because of compression after cutting, ensuring that sections lay flat on the slide to provide consistent immunostaining and image alignment. For meibomian gland ICT, BMMA embedded 2 year old mouse eyelid tissue was serially sectioned at 2 µm. Consecutive sections (156) were collected in ribbons which represented a total of 312 µm of tissue thickness (Fig. 6A). The mouse TG embedded in BMMA was sectioned at a thickness of 500 nm for greater axial resolution and a total of 40 serial sections were collected for a total reconstructed depth of 20 µm.

In the event of section loss or to remove sections with artefacts, defective image planes may be replaced with an interpolation of the plane in question with either adjacent intact plane. Interpolations can be achieved by merging or warping planes either in ImageJ or Photoshop and are averaged accordingly to fill in the absent data. The data set in this experiment required no such manipulation; however previous experience indicates that approximately 5% of sections may need such interpolation [21].

### Non-linear Optical and Confocal Microscopy

NLO microscopy was performed with the use of an ultra-short pulse Titanium-Sapphire femtosecond laser (Chameleon, Coherent, Santa Clara, CA). To map the collagenous extra-cellular matrix of the tarsal plate and eyelid (Fig. 6B), sections were excited using the femtosecond laser tuned to 800 nm and SHG signals from fibrillar collagen imaged using a Zeiss LSM 510 and a 20×/0.75 NA Zeiss apochromat objective. SHG signals were collected using the transmitted light detector with a 390/465 nm band pass filter and the Meta detector set to 380–420 nm [19], [20], [31], [32], [33], [34], [35]. These images were later used for segmentation and volumetric analysis of the meibomian gland, as detailed in our previous study [21]. After SHG imaging, sections were stripped of the BMMA polymer resin by acetone for immunostaining (Fig. 6C). To determine antibody penetration depth, confocal imaging of a CK6 immunostained 2 µm section from the 2month old mouse meibomian gland was performed using a 63×/1.4 NA Zeiss plan apochromat oil immersion objective with a step size of 0.1 µm.

### Immunostaining and Fluorescence Imaging

Ribbons of serial sections were encircled using an ImmEdge PAP pen (Vector Labs, Burlingame CA) so that solutions remained on each section at all times. BMMA is readily miscible in organic solvents and so it can be removed with washing in acetone for 10 minutes before rehydration in descending ethanol concentrations and finally, PBS. Next, heat-mediated antigen retrieval was carried out in a Pelco Biowave Pro 36500 laboratory microwave (Ted Pella) for 5 minutes using IHCTek Epitope Retrieval Solution (IHCWorld, MD). Each antibody staining step involved 6 minutes at 150 W, then 4 minutes at 0 W, before a final incubation at 150 W for 6 minutes in the Pelco microwave. Sections were then rinsed in PBS and the slides mounted in 50∶50 Glycerol:PBS with 1∶3000 4′-6-Diamidino-2-phenylindole (DAPI) to identify cell nuclei. All immunostaining steps were performed in the Pelco Biowave for rapid and consistent antigen labelling and sections between 0.5 µm and 5 µm were successfully stained.

Tissue sections were incubated in primary antibodies including rabbit polyclonal anti-CK1 (1∶2500 Abcam, Cambridge, UK), anti-Ki67 (1∶250 Abcam), anti-CK6 (1∶2500 Abcam), anti-CK10 (1∶2500 Abcam) and Alexa Fluor 546 Goat anti-rabbit secondary antibodies (1∶3000–1∶5000, Life technologies, Carlsbad CA), which were all diluted in 10% Goat serum in PBS as a blocking agent.

For the three-dimensional reconstruction, individual sections were imaged by non-linear optical microscopy before immunofluorescent staining using the Zeiss LSM 510 Meta and Zeiss 20×/0.75 NA objective. For detection of Alexa Fluor 546 fluorescence with the Zeiss LSM 510, a Helium-Neon 543 nm Laser and 565/615 band pass filter were used while DAPI stained nuclei were imaged separately using the chameleon laser tuned to 760 nm with a 435/485 band pass filter and the same 20x/0.75 NA objective. For each probe (SHG, DAPI, Ki67 and CK1), each section (n = 156) was tiled scanned (3×3 tiles) with a pixel size of 0.44 µm × 0.44 µm. Images were then stitched with a 10% overlap to form a 9 megapixel image covering an area of 1.274 mm × 1.287 mm. The individual montage images are then merged into a single 1.2 gigabyte sized stack containing the sequence of sections (4 files = 4.8 gigabytes). Between immunostaining steps, antibody probes were eluted using either glycine hydrochloride at a pH of 2.0 or 0.1% SDS, as previously described for paraffin sections [36]. Staining with the same secondary antibody (Alexa Fluor 546) illustrated the extent of antibody label removal (Fig. 6D) before being overlaid with NLO images (Fig. 6E) and further re-probing (Fig. 6F).

To evaluate antibody stripping and preservation of eYFP fluorescence in BMMA embedded tissues, 2month old mouse meibomian gland and eYFP-CD11c mouse TG tissue sections were imaged using a Nikon TE1000 epifluorescence microscope and a 60x/1.4 NA Nikon apoplan objective. Images were captured using a Photometrics CoolSnap FX camera (Roper Scientific, Tucson, AZ) and Meta Imaging series software (Molecular Devices, Downington, PA).

### Three-Dimensional Reconstruction

With each image acquisition, two channels of information were collected with one channel representing DAPI stained cell nuclei (i.e., DAPI and CK1). Thus, all data sets may be interleaved and co-aligned using the common DAPI channel. Image alignment and three-dimensional reconstruction of fibrillar collagen from SHG, as well as CK1 and Ki67 immunolabelling in the meibomian gland (Fig. 6G and H, respectively), was performed using the AlignSlices module in the Amira 5.4 software package (Visage Imaging, San Diego CA). For our reconstruction, we used a semi-automated alignment. For the reconstruction of an eYFP-CD11c^+^ cell in the mouse TG, 40 successive 500 nm sections were imaged with a 60x/1.4 NA Nikon objective at a pixel size of 0.11 µm × 0.11 µm and also aligned and reconstructed in Amira.

Segmentation of the meibomian gland was carried out using Adobe Photoshop CS5 and allowed for the calculation of whole gland and single acini (n = 10) volume based on voxel size. This involved marking the contours of the collagen fibres to manually segment the meibomian gland that is situated within the tarsal plate. Quantification of cell cycling was carried out using the ImageJ 3-D object counter plugin for DAPI stained nuclei and Ki67+ nuclei. To minimise over and under-counting of cell nuclei, 3-D nuclei counts were calculated over a range of threshold values and validated with physical counting. The threshold value (73) found to be the closest match to the physical counts was applied to each acini and the whole meibomian gland.

## Supporting Information

Movie S1 **Three-dimensional reconstruction of the collagenous tarsal plate (green) and segmentation of the meibomian gland for volumetric analysis.**
(AVI)

Movie S2 **Localisation of CK1 (yellow), cell nuclei (blue) and Ki67 (red) in the murine eyelid by ICT, which enables quantitative assessment of gland keratinisation, cell density and cell proliferation, respectively.**
(AVI)

Movie S3 **Three-dimensional reconstruction of the meibomian gland with CK1 (green), DAPI (blue) and Ki67 (red) labelling.**
(AVI)
